# An ABCG2 non-substrate anticancer agent FL118 targets drug-resistant cancer stem-like cells and overcomes treatment resistance of human pancreatic cancer

**DOI:** 10.1186/s13046-018-0899-8

**Published:** 2018-10-03

**Authors:** Xiang Ling, Wenjie Wu, Chuandong Fan, Chao Xu, Jianqun Liao, Laurie J. Rich, Ruea-Yea Huang, Elizabeth A. Repasky, Xinjiang Wang, Fengzhi Li

**Affiliations:** 1 Department of Pharmacology & Therapeutics, Roswell Park Comprehensive Cancer Center,Buffalo, New York, 14263 USA; 2 Canget BioTekpharma LLC Buffalo, New York, 14203 USA; 3 Center for Immunotherapy, Roswell Park Comprehensive Cancer Center, Buffalo, New York, 14263 USA; 4 Department of Immunology, Roswell Park Comprehensive Cancer Center, Buffalo, New York, 14263 USA

**Keywords:** FL118, Stem-like cancer cells, Survivin, Orthotopic pancreatic cancer model, Human pancreatic cancer patient-derived xenograft (PDX) animal models

## Abstract

**Background:**

Pancreatic cancer is a deadly disease with a very low 5-year patient survival rate of 6–8%. The major challenges of eliminating pancreatic cancer are treatment resistance and stromal barriers to optimal drug access within the tumor. Therefore, effective molecular targeting drugs with high intra-tumor access and retention are urgently needed for managing this devastating disease in the clinic.

**Methods:**

This study has used the following in vitro and in vivo techniques for the investigation of exceptional anticancer drug FL118’s efficacy in treatment of resistant pancreatic cancer: cell culture; immunoblotting analysis to test protein expression; DNA sub-G1 flow cytometry analyses to test cell death; MTT assay to test cell viability; pancreatic cancer stem cell assays (fluorescence microscopy tracing; matrigel assay; CD44-positive cell colony formation assay); human luciferase-labeled pancreatic tumor orthotopic animal model in vivo imaging; pancreatic cancer patient-derived xenograft (PDX) animal models; and toxicology studies with immune-competent BALB/cj mice and beagle dogs.

**Results:**

Our studies found that FL118 alone preferentially killed cisplatin-resistant cancer cells, while a combination of FL118 with cisplatin synergistically killed resistant pancreatic cancer cells and reduced spheroid formation of treatment-resistant pancreatic cancer stem-like cells. Furthermore, using in vivo-imaging, we found that FL118 in combination with cisplatin strongly inhibited both drug-resistant pancreatic xenograft tumor growth and metastasis. In PDX model, we demonstrated that FL118 alone effectively eliminated PDX tumors, while FL118 in combination with gemcitabine eliminated PDX tumors that showed relative resistance (less sensitivity) to treatment with FL118. These FL118 efficacy results are consistent with our molecular-targeting data showing that FL118 inhibited the expression of multiple antiapoptotic proteins (survivin, Mcl-1, XIAP, cIAP2) and ERCC6, a critical regulator of DNA repair, in treatment-resistant pancreatic stem-like cancer cells. Furthermore, FL118 toxicity studies in BALB/cj mice and beagle dogs indicated that FL118 exhibits favorable hematopoietic and biochemical toxicities.

**Conclusion:**

Together, our studies suggest that FL118 is a promising anticancer drug for further clinical development to effectively treat drug-resistant pancreatic cancer alone or in combination with other pancreatic cancer chemotherapeutic drugs.

## Background

Pancreatic ductal adenocarcinoma (PDAC) is the most common type of pancreatic cancer and is an extremely difficult-to-treat disease, causing ~ 40,000 deaths a year in the US with a dismal 5-year survival rate of 6–8% [1, 2]. In 2013, the FDA approved gemcitabine and Abraxane (albumin-bound paclitaxel) combination therapy as a first-line treatment for PDAC [3, 4]. Other combination treatment regimens include gemcitabine-cisplatin, gemcitabine-oxaliplatin and FOLFIRINOX (i.e. combination of 5-Fu, leucovorin, irinotecan, and oxaliplatin). FOLFIRINOX is used as an aggressive treatment only for carefully selected PDAC patients due to the serious toxicity of this regimen [5]. The poor therapeutic response of PDAC patients to treatment is due both to chemoresistance and impaired drug access to cancer cells mediated by desmoplasia [6, 7]. Therefore, development of novel drugs with favorable pharmacokinetic (PK) profiles of tumor accumulation to overcome chemoresistance and the desmoplasia barrier to drug access would fulfill an unmet need for effective treatment of PDAC.

We have identified a novel small antitumor molecule (FL118) through high throughput screening (HTS) of compound libraries by using cancer cell models in which the survivin gene acts as a biomarker and target [8], followed by in vitro and in vivo analyses of 207 available analogues of HTS-obtained top 20 hits [9]. FL118 was demonstrated to possess superb antitumor activity in human colorectal and head-&-neck cancer tumor animal models in comparison with clinically used drugs (irinotecan, topotecan, doxorubicin, 5-Fu, gemcitabine, docetaxel, oxaliplatin, cytoxan, cisplatin) [9]. While FL118 is structurally similar to camptothecin (CPT) and CPT analogues, irinotecan and topotecan (which are used in cancer treatment in the clinic), the anticancer efficacy of FL118 is irrelevant to the CPTs’ therapeutic target topoisomerase I (Top1) [10]. Instead, FL118 inhibits the expression of multiple cancer-associated antiapoptotic proteins (survivin, Mcl-1, XIAP, cIAP2) in colon, prostate and ovarian cancer cells [9]. Importantly, genetically forced expression or silencing of survivin, Mcl-1, XIAP, or cIAP2 in cancer cells, respectively, demonstrated their roles in mediating the action of FL118 [9, 11]. FL118 effectively overcomes irinotecan- and topotecan-resistant tumors in animal models [12]. ABCG2/BCRP [13–17] and MDR1/Pgp [18–21] are well-established efflux pumps for Top1 inhibitors irinotecan and topotecan, while FL118 is not a substrate for these drug-resistant factors [12, 22]. FL118 exhibits a favorable pharmacokinetics profile, accumulating and residing in tumors [12]. Since ABCG2 is a critical efflux pump that maintains viability of latent stem-like cancer cells [23–29], the capability of FL118 bypassing ABCG2 resistance makes it effective in killing latent, stem-like cancer cell populations and may also overcome the effect of desmoplasia-mediated limitation of drug access to pancreatic tumors. Therefore, development of novel drugs that can overcome ABCG2-mediated treatment resistance is one of the pressing issues in the field of anticancer drug development [30]. These characteristics of FL118 suggest that desmoplasia may not prevent FL118 from reaching therapeutic levels in the tumor microenvironment and within cancer cells. These observations therefore suggest that FL118 may have a great potential for effective therapeutics of treatment-resistant pancreatic cancer.

In this report, we present evidence that FL118 kills latent cancer stem-like cancer cells, inhibits tumor metastasis, and that FL118 alone or in combination with other pancreatic cancer cytotoxic drugs (e.g. gemcitabine, cisplatin) eliminates human pancreatic xenograft tumors in animal models. These findings are consistent with our observations that FL118 inhibits multiple antiapoptotic proteins (survivin, Mcl-1, XIAP, cIAP2) and critical DNA repair regulators such as ERCC6 in drug resistant pancreatic cancer cells. It is known that survivin [31–38], Mcl-1 [39–47], XIAP [35, 36, 38, 44, 48–55], and cIPA2 [55] are strongly involved in treatment resistance of PDAC; survivin [56–69] and Mcl-1 [70, 71] play important roles in cancer stem cell (CSC) drug resistance and function. ERCC6 is important for active gene repair [72], correcting transcription-coupled DNA repair defects [73] and being involved in drug resistance [74]. Thus, our study provides novel options for effective treatment of this devastating disease with FL118 alone or in combination with a pancreatic cancer drug that is currently being used for the treatment of pancreatic cancer in the clinic.

## Methods

### Cell lines, cell culture and reagents

The human pancreatic ductal adenocarcinoma cell lines PANC1, MIA PaCa2 (Mia2), B xPC3 and ovarian cancer cell line SKVO3 were originally obtained from ATCC. PANC1 that expresses luciferase (lucPANC1) was generated in this study with the parental PANC1 cell lines. Human ovarian cancer cell lines A2780 and its cisplatin-resistant counterpart A2780CP were gifts from Dr. Steven Howell [75]. All these cell lines were maintained in either DMEM or RPMI 1640 medium supplied with 10% fetal bovine serum (FBS, Atlanta Biologicals, Lawrenceville, GA), penicillin (100 units/mL) and streptomycin (0.1 μg/mL) (Invitrogen, Grand Island, NY). Cells were routinely subcultured twice a week and maintained in a humidified incubator with 5% CO2 at 37 °C. Cisplatin (Fresenius Kabi USA, LLC), gemcitabine (Pfizer) and Abraxane (Celgene) were from Roswell Park Comprehensive Cancer Center (Roswell Park) Hospital Pharmacy. Monoclonal anti-tubulin antibody, polyclonal anti-actin antibody and goat peroxidase-conjugated anti-rabbit IgG antibody were purchased from Sigma (St. Louis, MO). Antibodies for survivin (FL-142), ERCC1, ERCC6, γ-H2AX, ChK1, ChK2, ATM, ATR, RAD51, DNA Pol β and GAPDH were from Santa Cruz (Santa Cruz, CA). Antibodies for Mcl-1, XIAP, cIAP2, Bad, Bim, Bax, cleaved/activated caspase-3 and (cleaved and full length) PARP were from Cell Signaling (Beverly, MA, USA). MTT (3-[4,5-dimethylthiazol-2-yl]-2,5,-diphenyltetrazolium bromide) and leupeptin were purchased from USB (Cleveland, OH). MG132 was purchased from Medchemexpress (Princeton, NJ). D-luciferin potassium salt was purchased from Gold Biotechnology (St. Louis, MO). FL118 was synthesized in house with a purity ≥95% [11]. FL118 in the formulated suspension for in vivo animal oral administration is highly stable for more than 12 months and has no observable changes in its antitumor efficacy in comparison with the freshly prepared when tested in human tumor animal models.

### Western blot/immunoblotting analyses

Cancer cells treated with and without FL118 (in some experiments in the presence of MG132) were lysed in RIPA buffer containing 150 mM NaCl, 1.0% IGEPAL CA-630, 0.5% sodium deoxycholate, 0.1% SDS, and 50 mM Tris, pH 8.0. Fifty μg total protein from each sample were heated at 95 °C for 5 min after mixing with equal volume of 2X SDS loading buffer. Samples were separated on 12–15% SDS-polyacrylamide gel electrophoresis (SDS-PAGE) gels and electrotransferred to Pure Nitrocellulose Membranes (Bio-Rad, Hercules, CA). The membrane was then blocked in 5% skim milk in TBS-T buffer (20 mM Tris/HCl pH 7.5, 0.137 M NaCl, and 0.1% Tween 20) at room temperature for 2–3 h. Next, the membrane was incubated with different primary antibodies in TBS-T containing 5% BSA overnight at 4 °C in the range of dilutions from 1:500 to 1:2000. After washing with TBS-T, the membrane was incubated in TBS-T buffer containing 5% skim milk and corresponding secondary antibody (1:5000) for 45–60 min at room temperature with shaking. Protein of interest was detected using Western Lightning Plus –ECL (Perkin Elmer, Waltham, MA) and visualized by various times (3–120 s) of exposure. Actin was detected as the internal control to normalize total protein loading for each sample.

### DNA sub-G1 flow cytometry analyses

PANC1 and MIA PaCa-2 pancreatic cancer cells were treated with or without FL118 for 48 h, then harvested by trypsinization and washed with PBS. Cells (~ 1 × 10^6^) were resuspended in 5 mL 70% ethanol. After the initial fixation, cells were suspended in 0.5 mL PBS containing 25 μg/mL PI, 0.2% Triton X-100 and 40 μg/mL RNase A. After 30 min incubation at 4 °C, the cells were analyzed by flow cytometry. Data from flow cytometry was analyzed using WinList software (Verity Software House Inc., Topsham, ME) and presented as a relative fold sub-G1 DNA content increase in comparison with vehicle-treated controls. Triplicate assays were performed. The experimental results were expressed as the mean ± SD. The statistical significance of differences was determined by Student’s t-test between two groups.

### MTT assay

Cell growth/viability inhibitory effect of drug (FL118, gemcitabine, Abraxane, cisplatin) alone and in combination, respectively, on cell growth was determined by MTT cell viability assay. Viable cells (2500 cells per well) were plated in each well in 96-well plates. After an overnight incubation, cells were treated with and without relevant drugs alone and in combination, respectively, at various concentrations and incubated for 72 h. MTT, a colorimetric substrate, was added to a final concentration of 0.4 mg/mL to each well. Cells in 96-well plates were further incubated in a 5% CO_2_ incubator at 37 °C for 4 h, and then the medium was aspirated. The MTT metabolic product formazan was solubilized by adding 200ul of DMSO to each well. Absorbance in the relevant wells was measured at 570 nm using an Ultra Microplate Reader (Bio-Tek Instruments).

### Generation of A2780-GFPcODC, A2780CP-GFPcODC and PANC1GFPcODC cells and fluorescence microscopy to detect stem-like and drug resistant green cells

The retroviral expression vector pQCXIN-ZsGreen-cODC (ornithine decarboxylase), containing a green fluorescence marker, was kindly provided by Dr. Frank Pajonk [76]. The retroviral particles collected from packaging cells that transfected with the above vector were infected into A2780, A2780CP and PANC1 cells, respectively. The transfected cell pool was used for experiments. The transfected cells were treated with and without cisplatin or FL118 alone or in combination for defined times. Cell images were acquired digitally using the Olympus IX73 Inverted Scores (Olympus). Percentages of living cells were obtained from the average of the total cells counted from 10 microscopic fields.

### Matrigel stem cell cultural assay

The sphere formation assay was performed with PANC1 cells by treatment in the presence of vehicle and FL118 at 1, 10 and 100 nM. Spheres were maintained in Improved MEM containing 20 ng/mL epidermal growth factor, 10 ng/mL basic fibroblast growth factor, 5 μg/mL insulin and 0.4% Bovine Serum Albumin. PANC1cells were plated at a density of 1000 cells/well in 24-well ultralow attachment plates (Sigma-Aldrich). Briefly, 1000 PANC1 cells were suspended in 40 μL medium and mixed thoroughly with 60 μL BD Matrigel™ (BD Bioscience, San Jose, CA, USA). The mixture was plated onto the edge of the well, and the plates were incubated in a 5% CO2 incubator at 37 °C for 45 min to allow the BD Matrigel™ to solidify. Upon solidification, FL118 treatment was administered. Spheres in each well were set in the plate to grow for 20 days and then quantitated visually under a microscope at the magnification of 10X.

### CD44 positive cell sorting and CD44-positive cell colony formation

CD44 positive (CD44+) cells were purified (sorted out) using fluorescence activated cell sorting (FACS). FITC-conjugated mouse anti-human CD44 was used in FACS purification of CD44+ PANC1 cells. Briefly, PANC1 cells were harvested by using 0.25% trypsin/0.02% EDTA. After re-suspension of the cells in cell-culture media, the cells were counted and washed in PBS with 2% FBS and collected by centrifugation. Add 10 μL FITC-CD44 antibody to 1 mL PBS containing 1 million cells and 2% of FBS for 30 min on ice. Following the antibody labeling, PANC1 cells were washed in PBS with 2% BSA before flow cytometric analysis was carried out on a BD FACSAria™ III cell sorter (BD Science, USA). PANC1 CD44+ cells were plated in 6-well plates at the density of 300 cells/well, the cells were treated with vehicle or with FL118 at 1, 10 and 100 nM 24 h after seeding. Vehicle and FL118 were washed out with PBS after 72 h treatment; the cells were continuously cultured in complete medium with 10% serum in an incubator at 37 °C, 5% CO_2_ for 12 days. Colonies were fixed, stained with crystal violet solution and images were taken before counting colonies on day 12. The experimental results were expressed as the mean ± SD. The statistical significance of differences was determined by Student’s t-test between two groups.

### In vivo study approval

All in vivo experimental studies were performed following the IACUC-approved mouse protocol, which was approved by the Institutional Animal Care and Use Committee (IACUC) at Roswell Park Comprehensive Cancer Center.

### Human PDAC orthotopic tumor mouse model and treatment

PANC1 cells (**LucPANC1**) grown in culture medium were harvested by trypsinization, washed twice in ice-cold PBS, and adjusted to 5 × 10^7^ viable cells/mL. Twenty-μL volumes containing 1 million LucPANC1 cells were injected into a SCID mouse pancreas. Specifically, for intra-pancreatic implantation of LucPANC1 cells, SCID mice were anesthetized with isoflurane using the rodent anesthesia machine provided and maintained by the Animal Center. The surgical plane of anesthesia was monitored with pedal withdrawal reflex. Eye ointment was placed in the eyes. The abdomen was shaved and prepared using an iodine scrub and alcohol. An up to one cm incision was made using scissors, one third of the spleen was pulled out and LucPANC1 cells were injected in 20 μL volume (1 million cells) into the tail of the pancreas using a 29 gauge needle. The pancreas was returned to the abdomen and the abdominal wall musculature and the peritoneum were sutured using 5.0 absorbable surgical suture. The skin was closed with wet bond, which was removed 7 days after surgery. Postoperative analgesia was 0.05 mg/kg of buprenorphine subcutaneously q8–12 h postoperatively as needed. The mice were allowed to recover alone in a cage that was placed on a circulating warm water-heating pad. This reduces post-surgical shock. Once the mice were fully recovered from the surgical procedure and were freely moving around the cage with normal eating and drinking, the mice were returned to their cage. If any mice display signs of a moribund condition during the course of the study, they were euthanized.

Healthy orthotopic mice were randomly divided into 2 groups (3 mice per group): a control/vehicle group and an FL118-cisplatin treatment group (of note, LucPANC1 cells are drug resistant and in order to keep the amount of surgical mice to a minimum, the drug alone group was not included in the study). Treatments with vehicle and FL118 plus cisplatin were started 7 days after cell orthotopic implantation. The treatment schedule was that cisplatin at 5 mg/kg (half maximum tolerated dose, ~ 1/2MTD) and FL118 at 0.75 mg/kg (~ 1/6MTD via ip in our studies) were given on days 7, 14, 21 and 28 (q7d × 4) via intraperitoneal routes (ip). Bioluminescence imaging (BLI) was taken every 1–3 week (week 1 is the baseline).

### Mouse whole body in vivo imaging

SCID mouse whole body imaging was performed to detect luciferase reporter activity using the Xenogen IVIS® in vivo Imaging System (Caliper Life Science, Hopkinton, MA) in the Translational Imaging Shared Resource at Roswell Park. Prior to imaging, tumor-bearing mice were anesthetized with isoflurane (Patterson Logistics Services Inc., Mount Joy, PA) at 2.5% mixed with oxygen in a separate induction chamber. After induction anesthesia, mice were intraperitoneally injected with D-luciferin potassium salt dissolved in PBS at a dose of 75 mg/kg and transferred into the in vivo imaging chamber. Animals were provided 2.5% maintenance anesthesia within the imaging chamber, and ten minutes after D-luciferin injection BLI was performed for detection of luciferase activity. Following imaging, animals were returned to cages and monitored to ensure full recovery. Reported measurements of total flux, representative of BLI signal intensity, were obtained by tracing a region of interest over the entire tumor. All measurements and displayed pseudocolorized BLI radiance images were obtained using the Living Image (PerkinElmer, Waltham, MA) software.

### Human pancreatic cancer PDX tumor xenograft mouse model and treatment

Experiments followed the IACUC-approved mouse protocol. The human PDAC tumor xenograft animal work followed our previous protocols [9]. Briefly, human PDAC PDX tumors maintained on SCID mice were isolated, and a piece of non-necrotic tumor tissues (30-40 mg) were subcutaneously transplanted into the flank area of female SCID mice. Seven to 14 days after tumor transplantation at which PDAC PDX tumors were grown to150–250 mm^3^ (defined as day 0), mice were randomly divided into the required groups (5 mice per group) for FL118 alone or in combination testing. In this study, we used the timesaving intraperitoneal (ip) route for drug administration. The schedule for FL118 and gemcitabine is weekly for 4-time drug administration either alone or in combination. FL118 in the current study used a basic formulation recipe, which contains FL118 (0.1–0.25 mg/mL), DMSO (5%), and hydroxypropyl-β-cyclodextrin (0.05–0.125%, *w*/*v*) in saline. The formulation process was described in detail in the published patent (PCT/US2011/058558) [77]. The vehicle solution contains DMSO (5%), and hydroxypropyl-β-cyclodextrin (0.05–0.125%, w/v) in saline without FL118. Tumor length (L) and width (W) were measured using digital vernier calipers 1–3 times per week until the end of experimental studies. The tumor volume (v) was calculated using the formula: v = 0.5 (L x W^2^). Then the tumor size was divided by the day 0 tumor size as percentage tumor size versus day 0. The mean tumor volume ± standard deviation (SD) at each time point was derived from 5 mice in each group. The tumor curves were made using Sigma Plot software.

### Use of immune-competent BALB/cj mice for FL118 toxicity and MTD studies

Female BALB/cj mice at the age of six weeks were purchased from Jackson Laboratory. Mice were randomly divided into 4 groups with six mice per group. Mice were then treated with vehicle, FL118 at 10 mg/kg (this is the FL118 MTD in SCID mice), 12.5 mg/kg and 15 mg/kg via oral administration with the schedule qw × 4. MTD was defined as the highest dose that results in no drug-related moribund state or death, with a temporary body weight loss of no more than 20%, no significant clinical pathology changes including hematology and biochemistry parameters. Other signs of toxicity documented during the experiment included mouse behavior, fur status, movement and diarrhea. Mouse hematology and chemistry studies were performed in Roswell Park Animal Center in-house service using ProCyte Dx® Hematology Analyzer (IDEXX BioResearch) and Catalyst Dx® Chemistry Analyzer (IDEXX BioResearch).

### Use of immune-competent beagle dogs for FL118 toxicity and MTD studies

This is an outsourced study. The Contrast Research Organization (CRO) Covance has performed the studies. The experiment method in brief is below:

The FL118 MTD was estimated to be 12 mg/kg/dose for two doses in SCID mice. The calculated FL118 MTD for dogs is 2 mg/kg. Therefore, doses were set as 0.55, 1.1, and 2.2 mg/kg. Nine male and nine female purebred beagle dogs at the age of 5 months with a body weight range of 7.9–10.1 kg for male and 7.2 to 8.3 kg for females were received from Covance Research Products, Inc. (Cumberland, Virginia). Animals were acclimated to the test facility (Greenfield, Indiana) for 15 days prior to initiation. Animals were socially housed by sex. Animals were assigned to the study using a computerized procedure designed to achieve body weight balance with respect to group assignment with the following experimental design.

**Table:** 

Group^a^	No. of Animals		Dose Level (mg/kg/dose)	Dose Concentration (mg/mL)
	Male	Female		
1 (Vehicle control)	2	2	0	0
2 (Low dose)	2	2	0.55	0.11
3 (Mid dose)	2	2	1.1	0.22
4 (High dose)	2	2	2.2	0.44

^a^Group 1 received vehicle control article only

The formulation of FL118 in this study are FL118 at 0.11, 0.22 and 0.44 mg/mL in 0.44% (2-hydroxypropyl)-β-cyclodextrin (HPβCD), 2% hydroxypropyl methylcellulose (HPMC) and 1% propylene glycol (PG) in sterile saline (0.85% NaCl). This oral administration solution without FL118 was the vehicle control. Dose formulations were administered by oral gavage once on Days 1 and 8 of the dosing phase at a dose volume of 5 mL/kg. Doses were based on the most recently recorded scheduled body weight. Animals were checked twice daily (a.m. and p.m.) for mortality, abnormalities, and signs of pain or distress. Abnormal findings were recorded. Cageside observations were conducted for each animal once daily during the dosing phase, except on days when detailed observations were conducted. Abnormal findings were recorded. Detailed observations were conducted for each animal once during the predose phase and on Days 1, 4, and 8 (prior to dosing, as applicable) of the dosing phase. Detailed observations were also collected for each animal on the day of scheduled sacrifice. Abnormal findings or an indication of normal was recorded. On each day of dosing, cageside observations were conducted for each animal approximately 1, 4, and 24 h postdose. Abnormal findings were recorded. Postdose observation start times were based on the time dosing was completed for each group/sex. Body weights were recorded twice during the predose phase and on Days 1, 4, and 8 (prior to dosing, as applicable) of the dosing phase. Quantitative food consumption was recorded from Days 1 to 4, 4 to 8, and 8 to 9 of the dosing phase. Blood samples for hematology, coagulation, and clinical chemistry were collected from fasted animals via a jugular vein. Blood samples were collected once during the predose phase and on the day of scheduled sacrifice. The anticoagulants were sodium citrate for coagulation tests and potassium EDTA for hematology tests. Samples for clinical chemistry were collected without anticoagulant.

On Day 10 of the dosing phase, all animals, having been fasted overnight, were anesthetized with sodium pentobarbital, exsanguinated, and necropsied. Terminal body weights were recorded for sacrificed animals. A macroscopic examination of the external features of the carcass; external body orifices; abdominal, thoracic, and cranial cavities; organs; and tissues were performed. A Pathologist was available for consultation during necropsies. Organ weights were recorded at the scheduled sacrifice. Paired organs were weighed together. The statistical data analyses include means and standard deviations (SD) with the parameters of absolute body weight, body weight change, quantitative food consumption, continuous clinical pathology values, and terminal body weights and organ weights.

### Statistical analysis

The experimental data were analyzed using either Microsoft Excel or SigmaPlot and expressed as the mean ± SD. The statistical significance of differences was determined by Student’s *t*-test in two groups and one-way analysis of variance among multiple groups, with a *p*-value of 0.05 or less considering as significance.

## Results

### FL118 inhibits multiple antiapoptotic proteins in drug-resistant pancreatic cancer cells

**Fig. 1:**
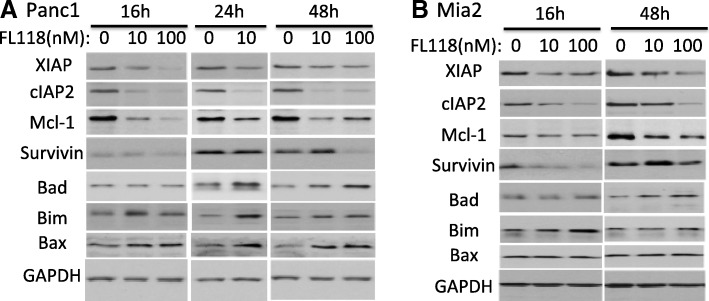
FL118 downregulates the expression of multiple antiapoptotic proteins and upregulates certain proapoptotic proteins in pancreatic cancer cells: Subconfluent pancreatic cancer cells were treated with FL118 as shown, and the expression of antiapoptotic proteins survivin, Mcl-1, XIAP, and cIAP2 as well as proapoptotic proteins Bad, Bim and Bax was detected by western blots using corresponding antibodies for each protein. GAPDH is an internal control for protein loading. The result from PANC1 pancreatic cancer cell line is shown in **a**, and the result from MIA PaCa2 (Mia2) pancreatic cancer cell line is shown in (**b**)

We have previously shown that FL118 is able to selectively inhibit the expression of multiple antiapoptotic proteins in colorectal cancer (CRC) cells [9]. We therefore determined whether FL118 also inhibits multiple antiapoptotic proteins (survivin, Mcl-1, XIAP, cIAP2) in treatment-resistant PDAC cell lines. Pancreatic cancer cell lines Mia-Paca2 (Mia2) and PANC1 were reported to be very aggressive and resistant to all first-line drugs [78], because of this we selected to use these cell lines to determine the effect of FL118 on the expression of various antiapoptotic proteins as well as some pro-apoptotic proteins using western blots. Our data showed that FL118 treatment in the range of 16 h (hours) to 48 h differentially modulates the expression of antiapoptotic proteins (XIAP, cIAP2, Mcl-1 or survivin) in both PANC1 and Mia2 cells in a dose-dependent manner (Fig. 1a and b), while such treatment induced the expression of pro-apoptotic proteins (Bad, Bim or Bax) in different degrees (Fig. 1a and b).

**Fig. 2:**
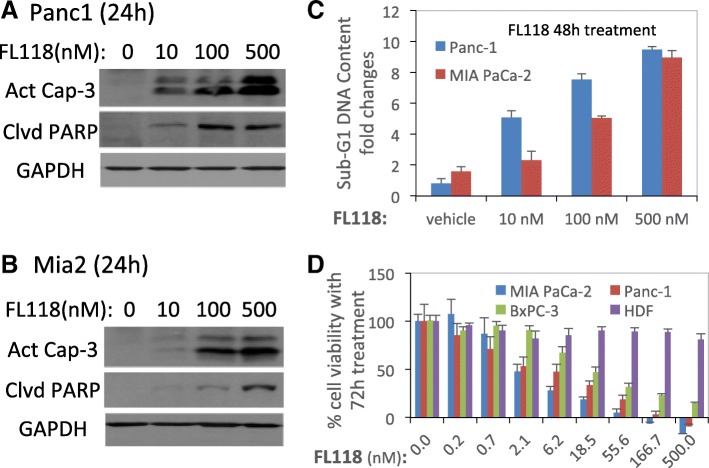
Induction of apoptosis, pancreatic cancer cell killing and cell viability inhibition by FL118: **a** and **b**, FL118 treatment results in activation of caspase-3 and cleavage of PARP. Subconfluent pancreatic cancer cells (A, PANC1; B, MIA PaCa2) were treated with FL118 as shown, and the activation of casepase-3 and cleavage of PARP were detected by western blots. GAPDG is the internal control for total protein loading. **c**, FL118 induces pancreatic cancer cell death. Subconfluent PANC1 and Mia2 pancreatic cancer cells were treated with vehicle or with FL118 at 10, 10 and 500 nM for 48 h. Then the sub-G1 DNA content (later apoptotic dead cells) was determined by flow cytometry. Relative sub-G1 DNA production levels were analyzed and the data were derived from 3 independent testing and shown as histogram as mean ± SD. **d**, FL118 inhibits pancreatic cancer cell viability. Subconfluent Mia2, PANC1 and BxPC3 pancreatic cancer cells as well as normal human dermal fibroblast cells were treated with vehicle (no FL118) or with a series of FL118 concentrations as shown for 72 h. Then the cell viability was determined using MTT assay. The data were shown as histogram with mean ± SD derived from 3 independent testing assays

Next, we determined whether FL118-mediated inhibition of antiapoptotic proteins and induction of pro-apoptotic proteins shown in Fig. 1 would be accompanied by induction of apoptosis and cell killing. Our studies indicated that as short as 24 h treatment with FL118 (10–500 nM) strongly induces caspase-3 activation and PARP cleavage (hallmarks of apoptosis, Fig. 2a, b) in PANC1 and Mia2 cells. Furthermore, the activation of these apoptotic markers was accompanied by a significant increase of sub-G1 DNA content in PANC1 and Mia2 cells, indicating the cell killing effect of FL118 (Fig. 2c). Consistent with these findings, we also determined the effect of FL118 on cell viability for PANC1, Mia2, as well as the gemcitabine-sensitive BxPC-3 pancreatic cancer cell lines. Our data revealed that FL118 at low concentrations (nM level) effectively inhibited pancreatic cancer cell viability (Fig. 2d). Based on the published status of gemcitabine resistance and sensitivity of PANC1, Mia2 and BxPC-3 [78], FL118 appeared to be more effective in inhibiting the viability of gemcitabine-resistant PANC1 and Mia2 cells in comparison with FL118’s effect on the viability of the gemcitabine-sensitive BxPC-3 cells (Fig. 2d). This observation is consistent with our previous finding that CRC cells with null or mutated p53 (which are resistant to DNA damage drugs) exhibit even higher sensitivity to FL118 treatment [79].

### FL118 sustainably inhibits ERCC6 and induces γ-H2AX in drug-resistant pancreatic cancer cells

**Fig. 3:**
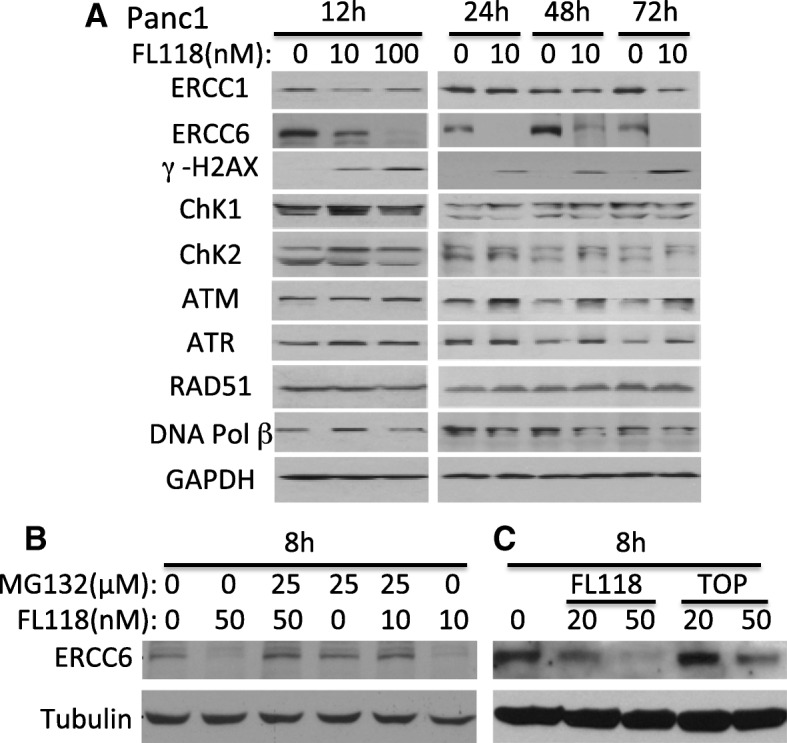
Effects of FL118 on the expression of proteins that are involved the pancreatic cancer cell DNA damage and repair: **a**, Subconfluent PANC1 pancreatic cancer cells were treated with FL118 as shown, and the expression of ERCC1, ERCC6, γ-H2AX, ChK1, ChK2, ATM, ATR, RAD51 and DNA Pol II was detected by western blots using corresponding antibodies for each protein. GAPDH is an internal control for protein loading. **b,** Decrease of ERCC6 expression can be rescued with proteasome inhibitor MG132. Subconfluent SKOV3 cells were treated with FL118 and MG132 alone or in combination as shown for 8 h, followed by western blot analyses with ERCC6 antibody. Tubulin is the internal control for protein loading. **c**, Comparison of FL118 and topotecan (TOP) effects on ERCC6 expression. Subconfluent SKOV3 cells were treated with FL118 or TOP as shown for 8 h, followed by western blot analyses with ERCC6 antibody. Tubulin is the internal control for protein loading

It is well known that drug/radiation-induced DNA damage can activate ATM signaling, which in turn results in the accumulation and activation of p53 and induces apoptosis. ATM has been reported to be able to directly phosphorylate p53 [80]. In contrast, our previous studies demonstrated that FL118-induced p53 accumulation is not mediated by the ATM signaling pathway, instead FL118 shifts the Mdm2-mediated ubiquitination and degradation of p53 (oncogenic signaling) to Mdm2-mediated ubiquitination and degradation of MdmX (apoptotic signaling) [79]. In order to have deeper insight into the relationship of FL118 treatment with potential cellular DNA damage, we determined the effect of FL118 on the expression of a panel of DNA damage and repair genes in the treatment-resistance pancreatic cancer cell line PANC1. We found that among the panel of proteins tested that are relevant to DNA damage/repair process (ERCC1, ERCC6, γ-H2AX, ChK1, ChK2, ATM, ATR, RAD51, DNA Pol β), FL118 strongly decreased the expression of ERCC6 and induced γ-H2AX (Fig. 3a). ERCC6 is a critical regulator for preferential repair of active genes [72, 73]. It has also been reported that elevated expression of ERCC6 confers resistance to 5-Fu treatment and is associated with poor survival in CRC patients [74]. Further studies indicated that inhibition of ERCC6 expression by FL118 appeared to be mediated by the proteasome degradation pathway since FL118-mediated downregulation of ERCC6 was rescued in the presence of the proteasome inhibitor MG132 (Fig. 3b). Additionally, FL118 had a much stronger effect than topotecan on the inhibition of ERCC6 expression (Fig. 3c).

### FL118 reduces the stem-like cancer cell population in drug-resistant ovarian and pancreatic cancer cells

**Fig. 4:**
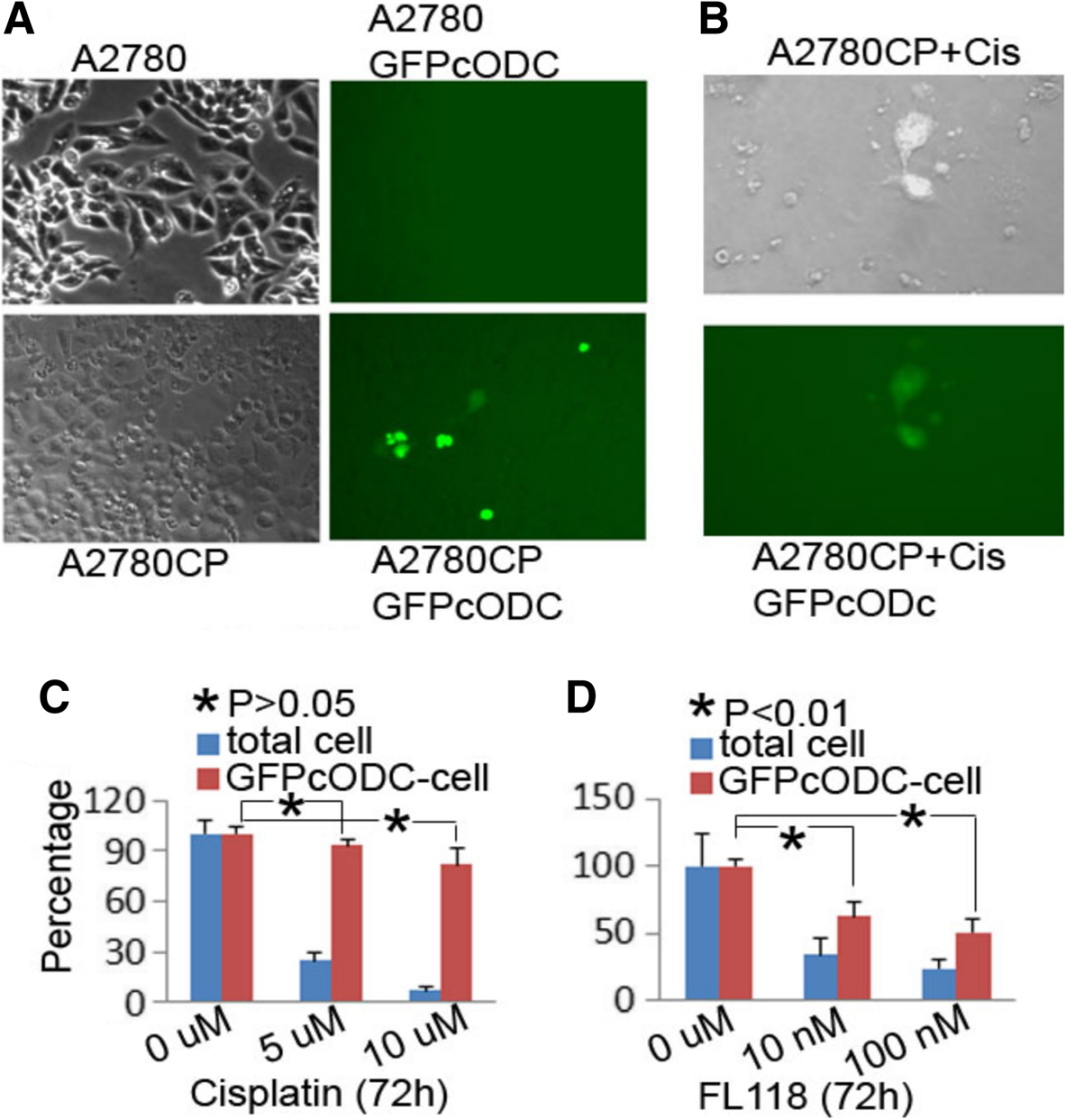
FL118 but not cisplatin is able to eliminate drug-resistant GFPcODC-positive A2780CP cells: **a** and **b**, Subconfluent cells were treated with and without cisplatin (Cis) for 72 h. Phase-contrast and GFP imaging of cells were digitally taken. **c** and **d**, Quantitative effects of cisplatin (**c**) and FL118 (**d**) treatment, respectively, on the percentage changes of drug-resistant GFPcODC positive A2780CP cells

Presented with the fact that FL118 targets and bypasses multi-drug resistance mechanisms, we hypothesized that FL118 should effectively reduce drug-resistant cancer cells including cancer stem cells (CSC) because the antiapoptotic proteins survivin [56–69] and Mcl-1 [70, 71] are known to play important roles in CSC drug resistance and function. To test this, we used a novel approach developed in Dr. Pajonk’s lab for the live tracking of drug resistant cells [76]. This approach takes advantage of reduced 26S proteasome activity of drug resistant cells. In the cell line that stably expresses the 26S activity reporter (GFPcODC fusion protein), only drug-resistant cancer cells will remain GFP-positive due to the reduced 26S activity [76]. This system was successfully applied to PDAC CSC tracking [81]. Here, we used both multidrug-resistant ovarian cancer cell line A2780CP [82] and drug-resistant PDAC cell line PANC1 [78] to test the above hypothesis. We found that in the established GFPcODC-expressing cells, there are significantly more GFPcODC positive cells in the drug-resistant A2780CP70-GFPcODC cells than in the parental drug-sensitive A2780-GFPcODC line (Fig. 4a). As shown, cisplatin treatment of A2780CP70-GFPcODC cells at 10 μM for 72 h killed 90% of the total cell population, but GFPcODC-positive cells survived (Fig. 4b, c). In contrast, FL118 treatment at 10 nM and 100 nM decreased more than 50% of the GFPcODC-positive drug-resistant A2780CP cell population (Fig. 4d).

**Fig. 5:**
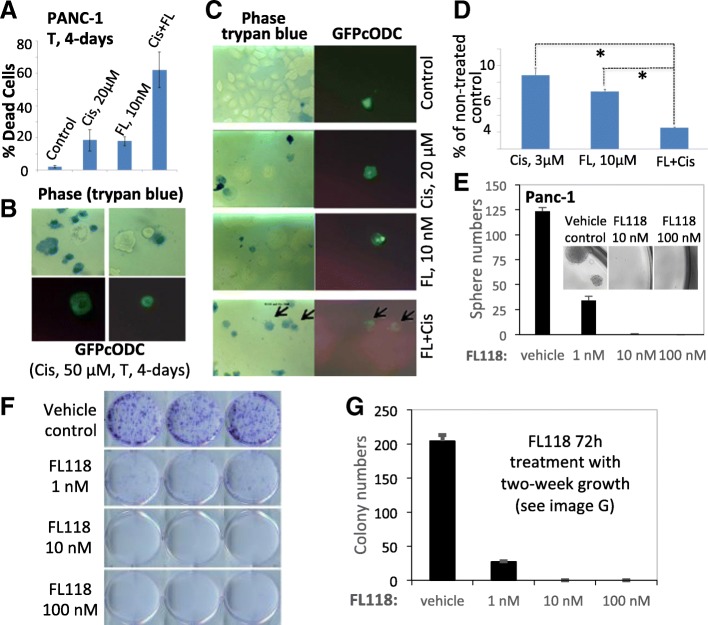
FL118 targets both proliferative cancer cells and latent stem cell-like cancer cells: **a**, FL118-cisplatin combination enhances killing of PANC1 cells. Dead cells were determined by trypan blue exclusion. **b**, GFPcODC-positive cells survive 50-□M cisplatin for 4 days, evaluated with trypan blue exclusion assay. **c**, FL118-cisplatin kills GFPcODC positive (drug-resistant) cells (arrows). Phase-contrast and GFP imaging of cells were digitally taken. **d**, FL118-cisplatin combination reduces PANC1 spheroid cell number. *, *P* < 0.001. FL, FL118; T, time. **e**, Effects of FL118 on pancreatic cancer stem cell sphere formation. See detail in the method section. **f**, Colony formation of CD44 stem cell marker-positive pancreatic cancer cells. **g**, Quantitative data derived from the data shown in the Fig. 5f

Next, we applied the same system to generate PANC1-GFPcODC cells. We tested FL118-cisplatin combination regimens for drug-resistant cancer cell killing measured by trypan blue exclusion assay. A 4-day single agent treatment with 20 μM cisplatin or 10 nM FL118 induced a similar degree of cell death (~ 18%), while combination treatment with FL118-cisplatin induced 62% cell death, indicating a 3-fold higher cell killing compared to the single agent treatment (Fig. 5a). GFPcODC-positive PANC1 cells were low (~ 0.5%) in PANC1-GFPcODC cell line but were extremely drug-resistant, which is consistent with the CSC feature in a typical cancer cell line. GFPcODC-positive (drug resistant) cells could survive 50 μM of cisplatin for a 4-day treatment, while the same treatment killed 92% of GFP-negative (drug sensitive) PANC1 cells (Fig. 5b). Interestingly, in this particular condition, single treatment with cisplatin at 20 μM, or with FL118 at 10 nM appeared unable to kill GFPcODC-positive (drug resistant) cells (Fig. 5c, Cis, 20 μM, FL, 10 nM), while the FL118-cisplatin combination killed the extremely drug resistant GFPcODC-positive PANC1 cells (Fig. 5c, FL + Cis, arrows). However, in our pancreatic CSC spheroid formation experiment, we found that most of the alive drug-resistant, stem cell-like cells were unable to form spheres (Fig. 5d). Specifically, in the spheroid formation for the 10-day pancreatic spheroid culture experiment, 10 nM FL118, 3 μM cisplatin, or their combinations were able to significantly inhibit spheroid formation, especially in the combination situation in comparison with no drug control (100, *p* < 0.001, Fig. 5d).

To further confirm these findings, we alternatively used the typical matrigel-based CSC sphere formation assay which is known to more closely mimic in vivo situations. In this assay only drug resistant stem-like cancer cells are able to form spheres. FL118 exhibited high efficacy in inhibiting sphere formation (Fig. 5e). To further demonstrate the effect of FL118 on stem-like, drug-resistant cancer cells, we isolated the CD44 stem cell marker-positive pancreatic cancer cell population from the drug-resistant PANC1 cells. We performed colony formation of CD44 stem cell marker-positive cells using the stem-cell culture condition. Consistent with the data from Fig. 5e, FL118 significantly inhibited colony formation at even 1 nM, while FL118 at 10 and 100 nM was able to completely eliminate colony formation of CD44 stem cell marker-positive PANC1 cell (Fig. 5f-g). These results (Fig. 5e-g) indicate that the drug-resistant, stem-like cancer cells in the CD44 stem cell marker-positive PANC1 cells are much more sensitive to FL118 treatment than the artificially-generated GFPcODC-positive PANC1 cells (Fig. 5c-d). Thus, the GFPcODC-positive cell model may be an extremely drug resistant cell model after transfection of the GFPcODC-expressing vector and selection process for obtaining GFPcODC-expressing cells, which may not fully mimic the CD44 stem cell marker-positive drug resistant cells. Alternatively, the discrepancy may also result from the further enrichment of the highly drug-resistant population after selection of GFPcODC-expressing cells. Together, our collective studies strongly suggest that FL118 alone and FL118 in combination with cytotoxic drugs are novel therapeutic options, which can effectively reduce drug-resistant, stem-like pancreatic cancer cell populations.

### Combination of FL118 and cisplatin inhibits both pancreatic cancer cell growth and tumor metastasis

**Fig. 6:**
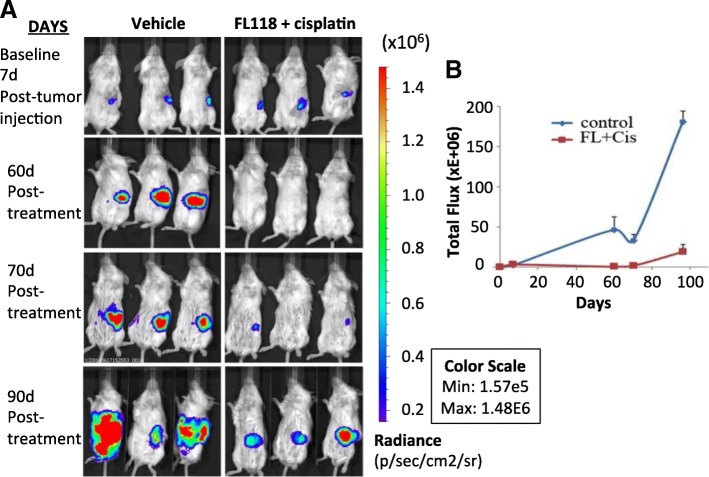
In vivo imaging to monitor pancreatic tumor growth and metastasis using an orthotopic LucPANC1 model: Several days after orthotopic implantation of LucPANA1 cells, mice were treated with and without FL118 and cisplatin as show via qw × 4 schedules. **a**, Bioluminescent images of whole mouse bodies treated with and without FL118 and cisplatin overtime (shown on days 7, 60, 70 and 90). **b**, Tumor growth curve of bioluminescent image (BLI) was shown as total flux overtime. d, day/days

PDAC resistance to treatment paves the way to cancer cell metastasis. Metastasis is a challenging concern since pancreatic cancer patients often present with metastatic disease [83]. To test whether FL118 could inhibit both PDAC tumor growth and metastasis to other sites, we used an orthotopic PANC1 cell model. In order to use bioluminescence in vivo imaging (BLI) to monitor tumor growth and metastasis in living mice, we generated luciferase expressing PANC1 cells (LucPANC1) and injected a million LucPANC1 cells in 20-μl volumes into the pancreas of SCID mice. These mice were treated either with vehicle (Fig. 6a, left panel, *n* = 3) or with FL118-cisplatin combination (Fig. 6a, right panel). These mice were treated with vehicle or FL118 at 0.75 mg/kg (1/6 maximum tolerate doses: 1/6MTD) in combination with cisplatin (5 mg/kg, 1/2MTD) via intraperitoneal (ip) injection from day 7, weekly for 4 times (qw × 4). BLI was performed every 1–3 week with 7d as the baseline (Fig. 6a, top panel). Our studies indicated that the radiance intensities of individual tumors fell in a very narrow range, suggesting a small variation of tumor implantation in our procedure (Fig. 6a). FL118-cisplatin treatment considerably inhibits PANC1 tumor growth and metastasis to other sites in the LucPANC1 orthotopic model in a relatively low dosing level. For example, on day 60 (60d) after LucPANC1 cell implantation, tumors in all treated mice were regressed to an undetectable level (Fig. 6a, second row). Of note, on day 90, after finishing treatment on day 28, BLI detected local metastasis in two of the three mice in the control group (4 red tumor centers and 2 red tumor centers, respectively), but no local metastasis were detected in the FL118-cisplatin-treated group (Fig. 6, 90d, forth row/bottom panel). Necropsy from the animals at the end of the study indicated the tumor not only on the pancreas (primary tumors), but also on the liver (one mouse with two sites and the other with one site) and small intestine (only the former mouse with one site), indicating tumor metastasis. In contrast, only significantly small tumors grew in the original tumor site (pancreas) of the FL118-cisplatin treated mice. These results indicate that our model system could precisely measure the onset of metastasis to other organ sites. The overall BLI intensity quantified from the total tumor of each group over time were presented in Fig. 6b. These observations indicate that FL118 is a unique and promising drug for killing both proliferative cancer cells and drug resistant/latent CSC-like cells, which will be further confirmed in the next experiment described below.

### FL118 eliminates human PDAC PDXs alone or in combination with gemcitabine in animal models

**Fig. 7:**
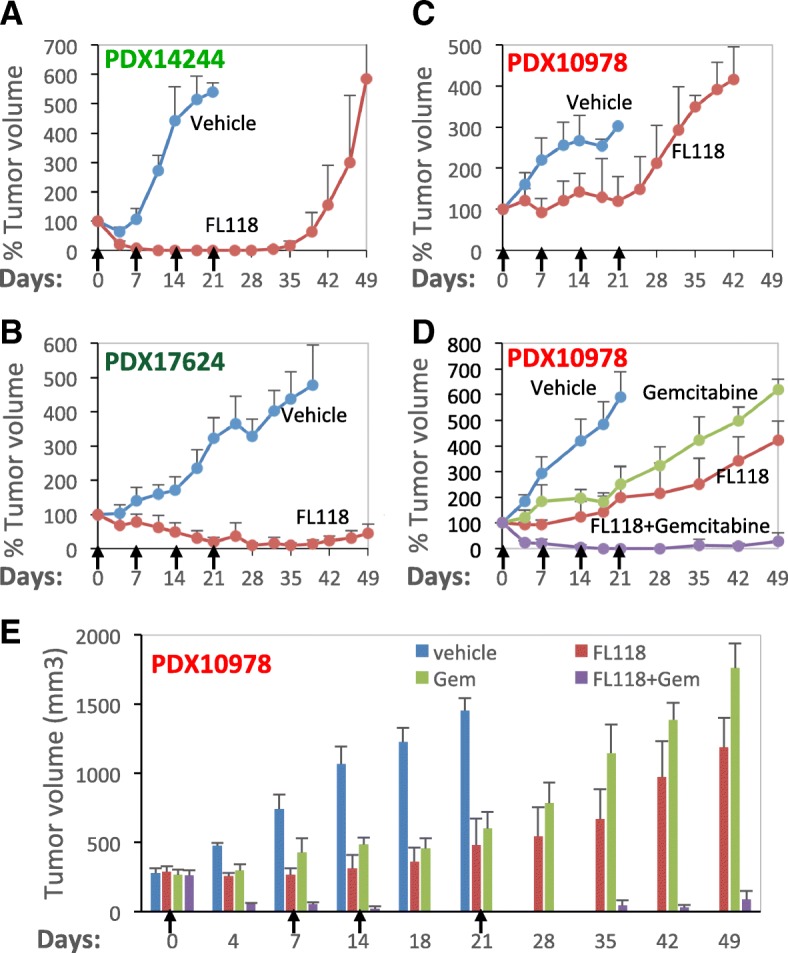
High efficacy of FL118 inhibition of pancreatic cancer PDX alone or in combination of gemcitabine: Pancreatic cancer PDX tumors were maintained on severe Combined Immunodeficiency (SCID) mice. Experimental SCID mice were subcutaneously implanted with individual pancreatic cancer PDX tumors at the flank area of mice. Seven to 14 days after the implanted tumors grew to 100–300 mm^3^ (designated as day 0), FL118 was administrated via ip with a dose of about half maximum tolerated dose (1/2MTD, 5 mg/kg) (**a**, **b**, **c**) or with a dose of 0.75 mg/kg in combination with gemcitabine at 60 mg/kg (~ 1/2MTD) (**d**) weekly for 4 times (qw × 4) as shown (arrows). **a,** Pancreatic cancer PDX14244 tumors. **b**, Results from pancreatic cancer PDX17624 tumors. **c** and **d**, Results from pancreatic cancer PDX10978 tumors. Each curve represents mean ± SD derived from five mice (5 mice per group). **e**, The same data shown in the Fig. 7d were presented in the tumor volume format to show the really size of the tumor during the experiments

Next, we used human pancreatic cancer PDX tumor models for alternative in vivo studies, since the PDX model is the most clinically relevant animal model and can present many of the histological features and heterogeneous treatment sensitivity/response of tumors found in patients [84, 85]. It has been recently reported that pancreatic cancer PDXs retain active paracrine/Hedgehog signaling, and desmoplasia is active in PDX tumor mouse models [86]. We have also shown that FL118 is not a substrate of ABCG2 and Pgp [12, 22], and exhibits a favorable PK (accumulating and residing in tumors after FL118 administration) [12]. Based on these characteristics of FL118, we reasoned that desmoplasia-mediated drug resistance might not be a barrier preventing FL118 from reaching therapeutic levels in cancer cells. Therefore, we tested the FL118 efficacy alone and in combination with gemcitabine in three human PDAC PDX tumor models. We found that both PDX14244 and PDX17624 showed high sensitivity to FL118, and FL118 was able to eliminate these PDX tumors only after one cycle of treatment (qw × 4, Fig. 7a, b). However, the third PDX tumor model, PDX10978 exhibited less sensitivity to FL118. As shown in Fig. 7c, FL118 was only able to delay PDX10978 tumor growth.

Next, we tested whether FL118 in combination with commonly used pancreatic cancer drugs such as gemcitabine could solve the FL118 insensitivity issue. We performed an FL118 and gemcitabine combination treatment on the FL118 less sensitive PDAC PDX10978 used above. In this study, we used FL118 at 0.75 mg/kg (~ 1/6MTD) and gemcitabine at 60 mg/kg (~ 1/2MTD) alone or in combination via ip with the qw × 4 schedule. We found that either FL118 or gemcitabine alone only delayed tumor growth. In contrast, a combination of FL118 with gemcitabine was able to eliminate PDX10978 tumors in all 5 treated animals for 21 days with only one cycle of treatment (Fig. 7d, from days 14–35). Alternatively, we used tumor absolute size histogram format to show the synergy of the combination treatment (Fig. 7e). Our studies suggest that a large percentage of PDAC tumors could exhibit high sensitivity to FL118, while some other PDAC tumors may show less sensitivity to FL118 treatment. However, PDAC tumors with less sensitivity to FL118 could be sensitized by FL118 in combination with a pancreatic cancer cytotoxic drug such as gemcitabine, used here to eliminate such PDAC tumors.

### FL118 exhibits favorable toxicology profile in both murine and canine animals

**Fig. 8:**
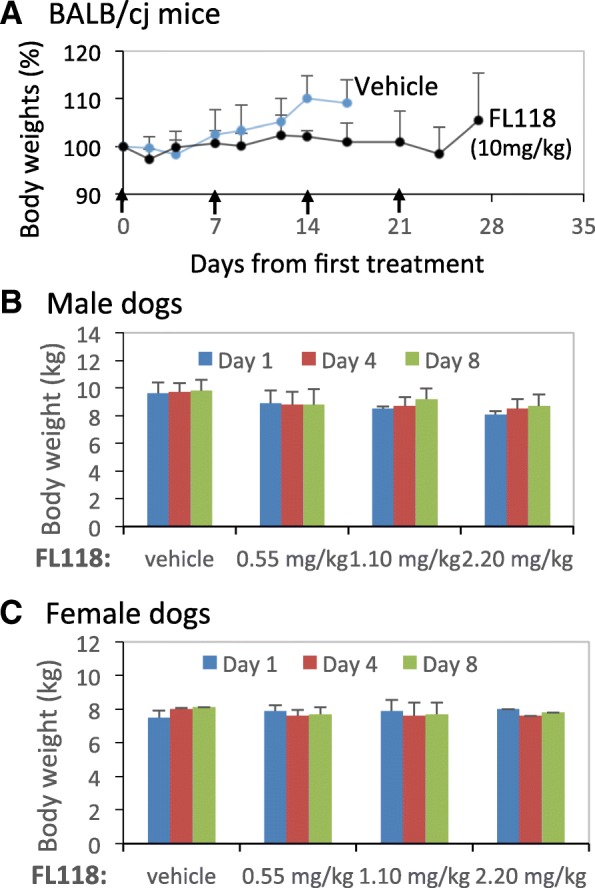
Effects of FL118 on animal body weight changes during treatment: **a**, Effects of FL118 on the body weight change of BALB/cj mice. Results from vehicle and FL118 MTD dosing level (10 mg/kg) were shown with oral route via qw × 4 (arrowed). The body weigh curves were derived from the mean ± SD derived from 6 independent BALB/cj mice. **b** and **c**, Effects of FL118 on the body weight change of beagle dogs. Results from vehicle and FL118 at 0.55 mg/kg (1/4MTD), 1.1 mg/kg (1/2MTD) and 2.2 mg/kg (MTD) dosing levels were shown with oral administration of FL118 on Days 1 and 8. The body weigh histograms were derived from the mean ± SD derived from 2 male or 2 female beagle dogs

Our studies indicated that FL118 in its oral formulation has an MTD at 10 mg/kg in human tumor-bearing SCID mice in the weekly for 4-time administration (qw × 4). Since SCID mice have deficient immune systems, it is very important to determine whether FL118 has a similar MTD in immune competent mice at the same route and schedule. We found that the use of ≥12.5 mg/kg oral qw × 4 resulted in > 20% body weight loss including some mice with moribund states. However, the use of 10 mg/kg was well tolerated by BALB/cj mice and the mouse body weight loss is within normal variation range (Fig. 8a). Consistently, the result from the evaluation of 17 hematological parameters indicated that only the white blood cells (WBC) and lymphocytes (LYMPH) decreased to the edge of normal range, low side (Table 1). After FL118 treatment, all others are similar to vehicle-treated samples, and within the normal range variation (Table 1). Similarly, among the 12 clinical chemistry parameters, the outcome from FL118-treated samples is very similar to the outcome from the vehicle-treated samples, which are close to or within the normal variation range (Table 2).

**Table 1:** Effects of FL118 on BALB/cj mouse hematological parameters

	**RBC** ^**a**^ **(M/μL)**	**HGB (g/dL)**	**HCT (%)**	**MCV (fL)**	**MCH (pg)**	**MCHC (g/dL)**	**RDW-SD (fL)**	**RET (K/μL)**	
Normal range	3.75–15.2	6.1–21.7	16.7–69.8	39–90.8	12.6–31	27–37.6	24.2–63.1	3.6–15.2	
Vehicle	7.51–9.06	11.1–13.7	33.5–40.6	44.6–44.2	14.8–15.1	33.1–34	26.4–28.9	5.13–6.4	
FL118 (MTD)	6.01–7.04	8.9–10.8	26.1–30.9	41.9–43.9	14.8–15.3	33.7–36	23–24.8	0.67–4.49	
	**PLT (K/μL)**	**PDW (fL)**	**MPV (fL)**	**WBC (K/μL)**	**NEUT (K/μL)**	**LYM (K/μL)**	**MONO (K/μL)**	**EOS (K/μL)**	**BASO (K/μL)**
Normal range	59–2633	5.7–23.9	5.2–13.1	1.1–56.1	0.03–32.1	0.12–23.5	0–5.1	0–2.1	0–2.3
Vehicle	179–290	7–11.2	5.5–7.6	4.0–8.5	3.6–5.7	0.31–1.73	0.26–0.95	0.01–0.12	0–0.01
FL118 (MTD)	487–1004	13.8–14.4	8.1–8.6	0.33–0.6	0.03–0.5	0.06–0.17	0.03–0.12	0.01–0.1	0

^a^ *RBC*, red blood cell, *HGB* hemoglobin, *HCT* hematocrit, *MCV*, *RBC* mean cell volume, *MCH*, mean corpuscular/cell hemoglobin, *MCHC* mean corpuscular/cell hemoglobin concentration, *RDW-SD* red cell distribution width-standard deviation, *RET* reticulocyte, *PLT* platelet, *PDW* platelet distribution width, *MPV* mean platelet volume, *WBC* white blood cell, *NEUT* neutrophil, *LYM* lymphocyte, *MONO* monocyte, *EOS* eosinophil, *BASO* basophil. M, million, *K* 1000/thousand

**Table 2:** Effects of FL118 on BALB/cj mouse serum biochemical parameters

	**GLU** ^**a**^ **(mg/dL)**	**BUN (mg/dL)**	**CREA (mg/dL)**	**PHOS (mg/dL)**	**Ca (mg/dL)**	**TP (g/dL)**
Normal range	90–192	18–29	0.2–0.8	6.1–10.1	5.9–9.4	3.6–6.6
Vehicle	89–140	8–15	≤ 0.1	4.6–5.5	9–10.8	3.9–4.6
FL118 (MTD)	87–185	16–19	≤ 0.1	10–13.3	8.1–9.4	3.4–4.1
	**ALB (g/dL)**	**ALT (U/L)**	**ALP (U/L)**	**TBIL (mg/dL)**	**CHOL (mg/dL)**	**AMYL (U/L)**
Normal range	2.5–4.8	28–132	62–209	0.1–0.9	36–96	1691–3615
Vehicle	1.9–2.1	76–124	42–82	≤ 0.1	112–114	1266–1272
FL118 (MTD)	1.7–2.2	33–58	52–105	≤0.1–0.3	91–109	1483–1982

^a^ *GLU*, glucose BUN/UN, (blood) urea nitrogen, *CREA* creatinine, *PHOS* phosphorus, *Ca* calcium, *TP* total protein, *ALB* albumin, *ALT* alanine transaminase/aminotransferase, *ALP/ALKP* alkalinephosphatase, *TBIL* total bilirubin, *CHOL* cholesterol, *AMYL* amylase

For the dog toxicology studies, all animals survived in good condition to the end of the experiment. No FL118-related clinical observations were noted. Certain observed fecal abnormalities were infrequent, transient, and noted for some animals during the predose phase; therefore, they were not FL118-related. No, or only minimal body weight changes within the variation of normal animal weight changes were observed for all FL118-treated groups (Fig. 8b, c). These observations are consistent with the outcomes from hematological analysis of the collected samples, most of which have a change within the pre-dosing variation. The results from vehicle and highest FL118 dose-treated dogs are shown in Table 3. As shown, in this FL118 MTD dose level, FL118 only exhibits very minor effects on a few hematological parameters such as decreased platelets and monocytes, but none of these are considered serious (Table 3). Similarly, in clinical chemistry studies, very few differences were present between control and FL118 test article-treated animals or between predose and dosing phase test results for individual dogs, and all were consistent with normal variation and considered incidental (Table 4). The observed differences were characterized by most or all of the following: small magnitude, no relationship to dose, inconsistent between sexes, absence of correlative findings, and/or similarity to differences present before initiation of dosing. Thus, overall the FL118 toxicology profiles in dogs are highly favorable, which is crucial as the physiology of dogs is much closer to humans than to the mice.

**Table 3:** Effects of FL118 on beagle dogs’ hematological parameters

	**RBC (M/μL)**	**HGB (g/dL)**	**HCT (%)**	**MCV (fL)**	**MCH (pg)**	**MCHC (g/dL)**	**RDW (%)**	**RET (K/μL)**	**PLT (K/μL)**	**WBC (K/μL)**
Vehicle TX										
pre-dosing	5.4–7.2	12.5–16.1	37.6–48.3	67–69.4	22–23.1	32.7–33.3	12.7–13.4	18.4–30.7	321–389	9.2–10.9
after dosing	6.0–6.7	13–14	39.4–44.3	66.3–68.7	21.7–23	32.8–34	12.6–13.3	14.1–34.5	256–283	9.8–14.1
FL118 (MTD)										
pre-dosing	5.1–5.9	11.8–13.2	35.4–40.2	67.4–69.3	22–23.2	33–33.5	13.4–13.4	11.6–45.3	318–386	7.1–8.7
after dosing	5.2–6.0	12–13.8	35.4–40	66–68.2	22.5–23	33.7–34.7	12.4–13.5	3.7–25.9	219–267	5.2–9.9
	**NEUT (K/μL)**	**LYM (K/μL)**	**MONO (K/μL)**	**EOS (K/μL)**	**BASO (K/μL)**	**LUC** ^**a**^ **(K/μL)**	**PT (sec)**	**APTT (sec)**	**FIB (mg/dL)**	
Vehicle TX										
pre-dosing	5.0–6.4	2.3–3.5	0.6–0.9	0.23–0.5	0.05–0.1	0.01–0.03	6.1–7.7	10.9–11.1	194–234	
after dosing	5.9–9.0	3.1–3.9	0.5–1.0	0.13–0.5	0.05–0.15	0.02–0.05	5.8–6.9	10.4–12	202–236	
FL118 (MTD)										
pre-dosing	3.7–5.2	2.4–3.7	0.5–0.6	0.18–0.26	0.05–0.1	0.02–0.05	6.1–6.9	10.5–11.7	209–313	
after dosing	3.2–9.0	1.6–3.0	0.1–0.41	0.06–0.28	0.01–0.03	0.00–0.01	5.6–6.4	10.1–11.2	210–364	

^a^ *LUC*, large unstained cells, *PT* prothrombin time, *APTT* activated partial thromboplastin time, *FIB* Fibrinogen

**Table 4:** Effects of FL118 on beagle dogs’ serum biochemical parameters

	**GLU (mg/dL)**	**BUN (mg/dL)**	**CREA (mg/dL)**	**TP (g/dL)**	**ALB (g/dL)**	**GLOB** ^**b**^ **(g/dL)**	**A:G Ratio**	**CHOL (mg/dL)**	**TRIG (mg/dL)**	**TBIL (mg/dL)**
Vehicle TX										
pre-dosing	68–91	9–13	0.2–0.4	4.7–5.2	3.2–3.6	1.5–1.7	1.9–2.3	133–160	37–48	≤ 0.1
after dosing	84–98	11–17	0.4	5.0–5.3	3.0–3.3	2.0–2.1	1.5–1.7	116–171	40–55	≤ 0.1
FL118 (MTD)										
pre-dosing	72–93	9–13	0.3–0.4	4.8–5.2	3.3–3.4	1.5–1.9	1.7–2.2	112–206	34–45	< 0.1
after dosing	87–105	12–20	0.4	4.7–5.2	2.8–3.2	1.8–2.1	1.5–1.8	119–195	18–46	≤ 0.1
	**AST (U/L)**	**ALT (U/L)**	**ALP (U/L)**	**GGT (U/L)**	**CK (U/L)**	**Ca (mg/dL)**	**PHOS (mg/dL)**	**Na (mmol/L)**	**K (mmol/L)**	**Cl (mmol/L)**
Vehicle TX										
pre-dosing	29–36	33–49	87–132	< 3	302–524	10.8–11.1	6.7–8.0	143–148	4.6–5.0	104–106
after dosing	34–88	41–46	96–129	< 3	387–4171	10.4–10.8	6.4–7.7	146–147	4.7–5.1	105–108
FL118 (MTD)										
pre-dosing	31–39	29–41	103–116	< 3	339–457	11–11.5	7.1–8.1	145–148	4.9–5.3	105–109
after dosing	40–48	23–51	81–106	< 3	410–794	10.2–10.7	6.1–7.0	146–148	4.5–4.9	108–112

^b^ *GLOB*, globulin *A:G* albumin:globulin, *TRIG* triglyceride, *AST* aspartate aminotransferase, *GGT* gamma glutamyl transferase, *CK* creatine kinase, *Ca* calcium, *Na* sodium, *K* potassium, *Cl* chloride

## Discussion

Our top goal in this study was to find out whether FL118 alone or in combination with common pancreatic cancer chemotherapeutic drugs used in the clinic could overcome treatment-resistance in pancreatic cancer. The basis for this potential, and the initiation of this study is that upregulation of antiapoptotic proteins, survivin [31–38], Mcl-1 [39–47], XIAP [35, 36, 38, 44, 48–55], and cIPA2 [55] are strongly involved in pancreatic cancer resistance, and that FL118 has been found to inhibit such gene expression in CRC and head-&-neck tumors [9]. Based on the known mechanisms of action (MOA) and other characteristics of FL118 [9], we first determined the effects of FL118 on the expression of both antiapoptotic and proapoptotic proteins in drug-resistant pancreatic cancer cells (PANC1, MIA PaCa-2). We found that FL118 downregulates antiapoptotic proteins and upregulates proapoptotic proteins (Fig. 1). However, the degree of modulation of the expression of these proteins by FL118 is dependent on cancer cell type, drug concentration and treatment time, which is consistent with our previous observations [9, 12]. Importantly, modulation of the expression of these proteins is associated with apoptosis induction, cell killing and cell viability inhibition in the drug-resistant pancreatic cancer cells (Fig. 2). Our previous studies indicated that FL118 treatment rapidly induced p53 accumulation via an ATM-independent signaling pathway [79]. In this report, we expanded our studies to determine the effects of FL118 on the expression of a panel of DNA damage and DNA repair-relevant proteins. We found that FL118 treatment induces a sustained inhibition of ERCC6 and induction of γ-H2AX (Fig. 3). This is a highly intriguing and novel finding, because it is well-documented that ERCC6 is important for active gene repair [72], correcting transcription-coupled DNA repair defects [73] and is involved in drug resistance [74]. The increase of DNA repair activity is known to contribute to PDAC resistance for commonly used pancreatic cancer drugs in the clinic [87–89]. In this regard, sustained inhibition of the critical DNA repair regulator ERCC6 may be one of the key mechanisms contributing to the anticancer efficacy of FL118 in the drug-resistant pancreatic cancer. This finding opens new perspectives and indicates an area for future studies. Alternatively, previous studies have suggested that increased H2AX expression may increase tumor sensitivity to chemo/radiotherapy in cancers [90]. Thus, sustained γ-H2AX induction by FL118 may also facilitate FL118 antitumor activity. Nevertheless, this FL118-induced sustained γ-H2AX expression is distinct from the transient induction of γ-H2AX resulted from DNA damaging drugs which attracts other proteins to form an γ-H2AX focus on the DNA double-strand break (DSB) site for efficient DNA repair with high-fidelity [91, 92]. This should facilitate DNA repair and thus should be a treatment resistant factor. In contrast, a sustained induction of γ-H2AX may have an opposing effect to increase FL118 effects on pancreatic cancer cell killing. This may indicate another new research area for future studies by use of FL118 as a tool to study the effects of FL118 on differential kinetic profiles of DNA damage versus DNA repair in relation to anticancer drug sensitivity versus resistance to pancreatic cancer.

Using a live tracking drug-resistant system (A2780GFPcODC versus A2780CP-GFPcODC), we demonstrated that FL118 preferentially reduces the cisplatin-resistant cancer cell population and kills GFPcODC-positive drug-resistant stem-like cancer cells (Fig. 4). We also generated the drug-resistant pancreatic cancer PANC1 cell model with the GFPcODC green marker and obtained the same result that FL118 in combination with cisplatin preferentially reduces the cisplatin-resistant PANC1 cell population and reduces treatment-resistant pancreatic cell spheroids (Fig. 5a, b, c and d). Consistent with the results obtained by using the artificial GFPcODC-transfected cancer cell model system, using the more physiological relevant matrigel stem cell sphere cultural condition (Fig. 5e) and stem cell marker CD44 selection (Fig. 5f, g), we further demonstrated that FL118 exhibited much higher effectiveness in inhibiting sphere and colony formation of pancreatic cancer stem-like cancer cells (Fig. 5e, f and g). This result suggests that FL118 may have high efficacy to inhibit both tumor cell proliferation and latent cancer stem cells for effectively eliminating pancreatic cancer in the condition more closely to the clinical practice situation. This is highly possible, because survivin [31–38], Mcl-1 [39–47], XIAP [35, 36, 38, 44, 48–55], and cIPA2 [55] play critical roles in pancreatic cancer resistance to treatment, and it is also known that survivin [56–69], Mcl-1 [70, 71] and XIAP [54] are involved in latent CSC drug resistance and function. Furthermore, it is well documented that ABCG2 as a critical efflux pump protein plays a vital role in maintaining viability of latent stem-like cancer cells [23–29], and the increase of ABCG2 [13–17], Pgp [18–21] expression is important for pancreatic cancer resistance to commonly used pancreatic cancer drugs in the clinic. In this regard, FL118 is not a substrate of these efflux pump proteins [12, 22] and can bypass these protein expression-induced treatment resistance [22]. Thus, FL118 has great potential to inhibit and eliminate drug treatment resistant pancreatic cancer in vivo due to the efflux pump protein overexpression.

In order to test the in vivo efficacy of FL118, we first determined FL118 inhibition of tumor growth and metastasis using orthotopic pancreatic tumor model. We stably transfected the luciferase reporter expression vector into PANC1 cells (LucPANC1) as a biomarker for bioluminescence in vivo imaging (BLI) and established orthotopic LucPANC1 tumor in SCID mouse pancreas. Our studies found that FL118 plus cisplatin can effectively eliminate pancreatic cancer cells and inhibit orthotopic pancreatic tumor growth and metastasis (Fig. 6). Thus, we established a good model for future studies regarding the effect of testing drugs on pancreatic tumor growth and metastasis inhibition.

Next, we alternatively used the current most-clinically relevant human pancreatic cancer model (pancreatic cancer patient-derived xenograft (PDX) tumor model) to test FL118 in vivo efficacy on pancreatic cancer tumor growth inhibition. We found that FL118 treatment can regress or eliminate some of pancreatic cancer PDX tumors (e.g. Figure 7a, b). However, for some other pancreatic cancer PDX tumors, FL118 exhibited much less sensitivity (e.g. Figure 7c). Significantly, pancreatic cancer PDX that exhibited less sensitivity to FL118 treatment could be eliminated by FL118 in combination with a pancreatic cancer drug such as gemcitabine (Fig. 7d). In order to have a better appreciation of the data shown in Fig. 7d, we presented this data in a tumor volume format (Fig. 7e). Based on the efficacy data seen in our PDAC PDX animal models treated with either FL118 alone or in combination with gemcitabine (Fig. 7), we predict that FL118 could be a highly promising anticancer drug for the effective treatment of drug resistant pancreatic cancer.

Based on these findings, a point of note is that if we could have an appropriate biomarker to stratify groups of patients: those with tumors that are highly sensitive to FL118 (e.g. these shown in Fig. 7a, b) and those with tumors less sensitive or even resistant to FL118 treatment (e.g. these shown in Fig. 7c); we can use FL118 as a monotherapy regimen for treating pancreatic cancer patients with tumors highly sensitive to FL118 treatment. In contrast, we can use FL118 in combination with a pancreatic cancer chemotherapeutic drug such as gemcitabine as a combination regimen to treat pancreatic cancer patients with tumors less sensitive or even resistant to FL118 treatment. This would aid in identifying patients who could benefit from FL118 as a monotherapy regimen (to avoid unnecessary combination treatment), and those who may need FL118 in combination with gemcitabine or Abraxane. This is ethically and economically significant because it could help to avoid unnecessary drug toxicity and save on medical expenses. We are currently in efforts to characterize several potential FL118 biomarkers. One option that can be disclosed at this time is the Kras mutation as a biomarker (Sreevidya Santha, publication in preparation), since Kras mutation occurs in over 90% of the pancreatic cancer. Our preliminary data indicated that pancreatic cancer with a Kras mutation exhibits high resistance to common cytotoxic drugs but more sensitivity to FL118. This is consistent with our previous studies that the more malignant a CRC tumor, the more effective FL118 treatment will be. For example, we found that while FL118 induced cancer cell senescence in CRC cells with wild type p53, FL118 exhibited higher activity to induce CRC cell death in CRC cells without a functional p53 [79].

FL118 toxicology studies in both BALB/cj mice and beagle dogs resulted in a highly favorable toxicology profile for FL118, even at the FL118 MTD dosing levels (Fig. 8 and Tables 1, 2, 3 and 4). Given that FL118 exhibited high efficacy to eliminate drug-resistant pancreatic cancer PDX tumors alone or in combination with gemcitabine (Fig. 7) and that relapse head-&-neck and CRC xenograft tumors are still highly sensitive to FL118 re-treatment [12], the favorable toxicology profile of FL118 would lay a foundation for further development of FL118 through clinical trials for treatment of patients with advanced and drug-resistant pancreatic cancers.

## Conclusions

Our studies indicated that FL118 exhibited a favorable toxicology profile in both BALB/cj mice and beagle dogs, FL118 inhibits and/or bypasses multiple pancreatic cancer-associated treatment resistant mechanisms and has high efficacy to eliminate drug-resistant pancreatic cancer PDX tumors alone or in combination with gemcitabine. Thus, FL118 is a promising anticancer drug to be further developed for effective treatment of PDAC either alone (monotherapy) or in combination with commonly used clinic cytotoxic drugs such as gemcitabine, Abraxane or cisplatin. Growing evidence indicates that FL118 has the potential to be a great platform to be used for generating FL118 derivatives for human disease treatment [93, 94].

## Abbreviations

BLI: Bioluminescence imaging
CI: Combination index
CPT: Camptothecin
CRC: Colorectal cancer
DAPI: 4,6-diamidino-2-phenylindole
MTD: Maximum tolerated dose
MTT: 3-[4,5-dimethylthiazol-2-yl]-2,5,-diphenyltetrazolium bromide
PDAC: Pancreatic ductal adenocarcinoma
PDX: Patient-derived xenograft
PI: Propidium iodide
PK: Pharmacokinetics
qw: Weekly
SCID: Severe combined immunodeficiency
SD: Standard deviation
SDS: Sodium dodecyl sulfate
Top1: Topoisomerase 1
